# Design of adaptive hybrid MPPT controllers with universal input voltage DC–DC converter for RES’s

**DOI:** 10.1038/s41598-024-62208-7

**Published:** 2024-05-18

**Authors:** Shaik. Rafikiran, Faisal Alsaif

**Affiliations:** 1 Sri Venkateshwara College of Engineering (Autonomous), Tirupati, AP 517502 India; 2 Department of Electrical Engineering, College of Engineering, King Saud University, 11421 Riyadh, Saudi Arabia

**Keywords:** Duty signal, Efficiency of the MPPT controller, Fast fuel stack response, High voltage gain, Plus low voltage appears across the switches, Engineering, Electrical and electronic engineering

## Abstract

At present, conventional energy production is absent because of the more hazardous gases released into the environment, the high effect on human health, more cost required for maintenance, plus less usefulness for highly populated areas. So, the Renewable Energy Sources are more focused for the present automotive industry application. In this work, the Proton Exchange Membrane Fuel Stack is considered for analyzing the proposed DC–DC converter circuit. The advantages of this fuel stack are high energy density, fast functioning nature, more robustness, and more usefulness for the various water membrane conditions of the fuel stack. However, the disadvantages of the fuel stack are excessive current generation, plus more current conduction losses. So, the wide voltage supply single switch power converter is introduced in this work for optimizing the current production of the fuel stack network. The merits of this converter circuit are high stability, good reliability, low voltage appearing across the switches, plus a uniform power supply. Here, the converter switching pulses are obtained by proposing the Modified Continuous Step Change Adaptive Fuzzy Logic with Grey Wolf Optimization hybrid controller. This controller provides high maximum power extraction efficiency from the fuel stack which is equal to 99.421%. Also, this controller's Maximum Power Point Tracking time is 0.0285 s.

## Introduction

From the literature study, conventional power networks are more harmful to human life and release high-level greenhouse gas emissions in nature. Also, this source is not flexible for all locations to install. So, researchers are moving towards renewable systems for producing electrical energy. The present utilized renewable systems are tidal, geothermal, solar, biomass, and wind power production networks. In^1^, the researchers discussed the tidal power technology for electricity production to the coastal area power consumption. Tidal energy production varies from one season to another season, and it is a nonuniform power supply category. Also, the tidal network power conversion efficiency is very low when associated with thermal power networks. The disadvantages of tidal power networks are more initial construction price, more complexity in setting the tidal power blades near the dam, applicable for very few places, and high effect on wildlife animals, and plants^2^. So, the geothermal energy networks are located in most of the local places for efficient energy production for small-scale industry applications. In nature, the geothermal supply availability is less. In addition, the demerits of geothermal networks are the high generation of waste, causing earthquakes, and more upfront costs when associated with tidal energy networks^3^.

The geothermal drawbacks are overcome by utilizing the bioenergy system. In this system, the biogas is heated thereby producing electrical energy. Here, the running of the turbine spins coupled with the biogas-dependent generator for functioning the entire system^4^. The advantages of a bioenergy system are less cost for energy production when associated with other fossil fuels, the most environment-friendly nature, more abundant, and highly reliable. However, it has the drawbacks of more space required, the possibility of greenhouse emissions, and moderate expense. In^5^, the authors suggested the hydro networks for energy production to the standalone network. Here, the H_2_O kinetic energy is transferred into rotational kinetic energy for moving the hydro turbine thereby rotating the three-phase generator. The merits of these hydro systems are inexpensive, ability to produce locally, are more useful for irrigation systems, ability to help under peak load conditions, and are very well paired with the other renewable energy systems. However, the disadvantages of this network are required high up-front cost, less availability of reservoirs, and moderate effect on the atmospheric conditions.

In^6^, the researchers used the sunlight topology for a smart grid hybrid power network to enhance consumer power utilization reliability. The sunlight topology supplies the energy to the microgrid under emergency conditions for maintaining continuous power to the consumers. This system's energy supply depends on the diode operation. In the silicon diode, the free electron consumes the sunlight intensity. As a result, the free available electron moves from an n-type device to a p-type device for the production of electricity. One-cell power utilization is not possible^7^. So, the multiple numbers of cells are intercompared for reaching the required peak power rating. As of now, in the market, there are three main types of PV networks available in the literature based on the application which are on-grid, off-grid, and hybrid networks. Here, each network has its own merits and disadvantages. The PV cells are manufactured by using crystalline silicon, single crystalline, multiple types of crystalline, and thin film technology. Now, most of the power generation corporations are using single crystalline devices for implementing the PV system because of the high sunlight energy conversion efficiency, and more flexibility^8^. However, the sunlight network provides nonuniform energy which does not apply to the electric vehicle network. The drawbacks of the above renewable power networks are compensated by considering the fuel stack technology. This methodology provides energy until the source H_2_ is supplied.

**Figure 1:**
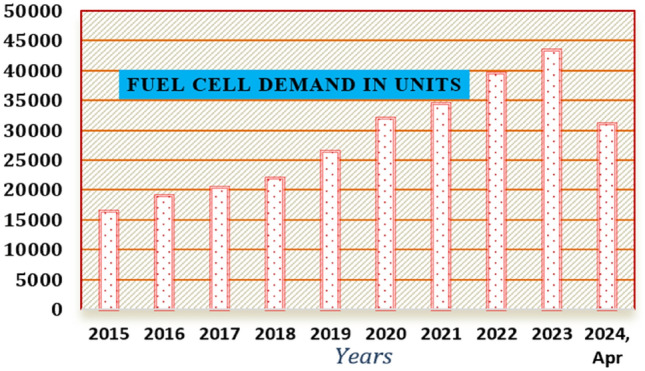
Fuel cell utilization from one state to another stage^9^.

In^9^, the authors referred to the solid oxide electrode-dependent fuel stack for running heavy vehicles with adjustable load speed. This type of fuel cell is more popularly used for stationary 100 W to 2 MW power range applications. The functioning temperature of this fuel stack is 500–1000 °C, and its power supply efficiency is 50–60%. The electrolyte manufacturing of this fuel stack has been done by considering stabilized zirconia and ceramic matrix with free electrons. For this fuel cell, natural gas, propane, methane, and hydrocarbons are used as the inputs for the continuous energy supply to the hospital. Finally, the operational life cycle of this fuel stack is 40,000 h. However, this fuel network has the drawbacks of more starting time because of the high operating temperature, and excessive chemical and mechanical compatibility issues^10^. So, the problems of solid oxide technology are compensated by using the alkaline cell. Its functioning temperature capacity is 100 °C and can give 55% power conversion efficiency. In this stack, the aqueous solution-based potassium hydroxide-soaked act as an electrolyte. For this fuel stack, the pure H_2_ is supplied to the input of the alkaline cell. The demerits of this controller are more cost and limited running lifetime and its demand outside of the market is represented in Fig. 1.

In^11^, the authors work on the phosphoric acid-dependent fuel network for submarine application because of its high ability to operate at low-temperature values, and heat conversion efficiency in the fuel stack is 90%. However, this fuel stack has the drawback of low power conversion efficiency which is equal to 42.45%, and it works up to 160 °C temperature conditions. The electrode material used in this fuel stack is pure phosphoric acid, and the selected supply fuels are natural gas and H_2_. The features of this stack are high tolerance for the other unwanted chemicals that exist in nature^12^. However, the problem with this method is the high design cost because the usage of high-cost platinum is involved in the catalyst. In this article, the polymer membrane material-based fuel stack is recommended for the energy supplied to hydrogen-dependent vehicles to mitigate the issues of other existing fuel cells. However, the problem of this cell is the nonlinear energy supply to the consumers. As a result, it creates heating losses in the overall hydrogen vehicle network. So, the peak power extraction methodologies are investigated in^13^ for maximizing the power generated from the fuel stack and maintaining the uniform energy to the consumer.

The more causally used peak power point identifying methodology is Perturb & Observe (P&O) which is useful for the traffic light controlling system. This is very easy to develop because of the low number of sensing devices selected for optimizing the power distribution losses of the automotive system. However, it produces a greater number of disturbances in the battery-based microgrid network^14^. The microgrid is interconnected with the solar, interleaved Y-source power converter, and charging station. In this microgrid, P&O controls the battery state charging conditions as well as discharging state conditions. The working efficiency of the P&O method in this smart grid network is very low which is enhanced by using the Incremental Conductance (IC) technology^15^. This methodology provides more efficient power to the hydrogen vehicle system and gives a good dynamic system response. Due to the high implementation cost of this controller, it is not applicable for street light conditioning networks. So, in this work, the modified continuous step changes adaptive fuzzy logic with the grey wolf optimization concept is introduced for optimizing the power oscillations of the fuel cell network. The features of this controller block are good flexibility, good tracking response, high robustness, and quick adaptability with the change of temperature conditions of the fuel stack^16^.

One more issue of fuel stack is excessive supply current with less potential. Due to this feature, the hydrogen fuel stack is not directly feeding to the automobile system. So, the converter technology is selected in the hydrogen fuel system for reducing the output current flow of the system thereby enhancing the consumer load voltage. From the literature review, the converter technologies are transformer-oriented devices and non-isolated converter devices. In^17^, the transformer-oriented device is used for the islanded grid-connected network to improve the load voltage profile. Here, the Z-source external circuit is integrated with the existing converter circuit to enhance the voltage conversion ratio of the hydrogen fuel network^18^. However, this type of circuit takes a high amount of cost, needs more passive components for manufacturing the circuit, more external circuits are needed, and is less robust. So, the Single Switch Wide Voltage Supply Power Converter (SSWVSPC) topology is introduced in this work to increase the potential value of the hydrogen fuel stack network. The advantages of this network are better dynamic behavior, more voltage conversion efficiency, low-level voltages appearing across the switches, and good reliability.

## Mathematical analysis of fuel stack dynamics

**Figure 2:**
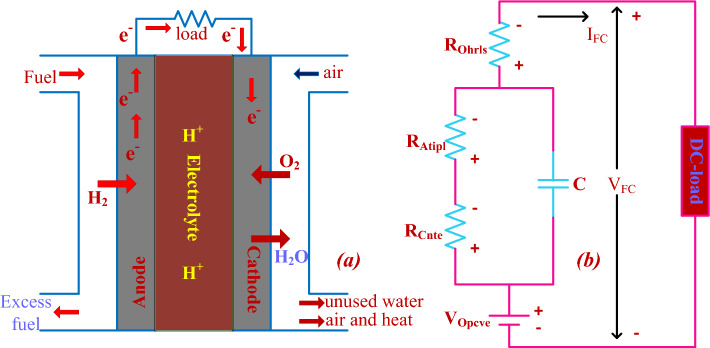
(**a**) Working states of the fuel stack network, and (**b**) its electric current flow circuit.

Fuel stack thermodynamic analysis is the most powerful idea for running the hydrogen system at various temperature values. From the previously available articles, the molten carbonate electrolyte material-based hydrogen fuel cell's maximum efficiency is 38%, and its functioning temperature range is between 558 and 658 °C^19^. The main application of this fuel stack is suitable for high-power rating standalone applications. The merits of this fuel stack technology are more suitable for any hybrid power supply application, has more energy density, is atmospheric friendly, and is more robust. However, the demerits of molten carbonate technology are highly expensive and more time-consuming for stabilizing the fuel production power^20^. So, all those drawbacks of the molten carbonate electrolyte system are limited by using the polymer membrane electrolyte in the proposed fuel stack network. The functioning structure of the polymer-based cell is discussed in Fig. 2a, and its current formation is given in Fig. 2b. From Fig. 2a, the H_2_ hydrogen gas is fed to the left side of the cell, and the right side is fed with natural gas. The combination of the oxygen from the cathode, and hydrogen from the anode forms the water, and electrical powers are the byproducts.

Here, the voltage that appeared across the cell under the circuit open condition is V_Opcve_, and its closed-circuit voltage and currents are named V_FC_, and I_FC_. At the time of fuel stack operation, the reversal currents passing through the electrolyte are I_Cnte_, I_Atipl_, and I_Ohrls_, and its corresponding concentrative, ohmic, plus active voltages are identified as V_Cnte_, V_Atipl_, and V_Ohrls_. The rate of chemical reaction in the cell is derived in Eq. (3). From Eq. (3), the pure hydrogen is combined with the air to form the water in the power production network. The obtained useful energy from the fuel stack is connected to the consumer load through the power converter circuit^21^. The entire cells utilized in the fuel network are represented as “N” and its associated produced voltage is equal to V_Overall_. At fuel network functioning temperature (T_Fsft_), the available fuel stack open-circuited voltage is discussed in Eq. (6). In any fuel stack device, there are two pressures have existed in the system which are hydrogen partial pressure (${\text{P}}_{{{\text{H}}_{2} }} )$ plus oxygen partial pressure. Similarly, the anode layer humidity and cathode layer humidity constants are identified as H_anode_, plus H_Cathode_ and its associated pressures are P_anode_, plus P_Cathode_ respectively.

1 $${\text{H}}_{2} \;\left( {{\text{Hydrogen}}} \right) \Rightarrow 2{\text{H}}^{ + } + 2{\text{e}}^{ - }$$

2 $$2{\text{H}}^{ + } + 2{\text{e}}^{ - } + \frac{1}{2}{\text{O}}_{2} \Rightarrow {\text{H}}_{2} {\text{O }}\left( {{\text{Water}}} \right)$$

3 $${\text{H}}_{2} \;{\text{hydrogen}} + \frac{1}{2}{\text{O}}_{2} \Rightarrow {\text{H}}_{2} {\text{O }}\left( {{\text{water}}} \right) + {\text{Useful }}\;{\text{Energy}}$$

4 $${\text{Overall }}\;{\text{stack }}\;{\text{avaialble}}\;{\text{ enetgy }}\;{\text{V}}_{{{\text{Overall}}}} = {\text{N*V}}_{{{\text{FC}}}} { }\left( {\text{each cell}} \right)$$

5 $${\text{each }}\;{\text{cell }}\;{\text{V}}_{{{\text{FC}}}} = {\text{V}}_{{{\text{Opcev}}}} - {\text{V}}_{{{\text{Cnte}}}} - {\text{V}}_{{{\text{atipl}}}} - {\text{V}}_{{{\text{Ohrls}}}}$$

6 $${\text{V}}_{{{\text{Opcev}}}} = 1.27321 - 0.07954{\text{e}}^{ - 2} \left( {{\text{T}}_{{{\text{Fsft}}}} - 297.998} \right) + {{\S}}$$

7 $${{\S}} = 43.22*{\text{e}}^{ - 6} \times \log ({\text{P}}_{{{\text{H}}_{2} }} \sqrt {{\text{P}}_{{{\text{O}}_{2} }} } ){\text{*T}}_{{{\text{Fsft}}}}$$

8 $${\text{partial }}\;{\text{pressure }}\;{\text{P}}_{{{\text{H}}_{2} }} = 0.5{\text{ H}}_{{{\text{anode}}}} {\text{P}}_{{{\text{H}}_{2} {\text{O}}}}^{{{\text{sat}}}} { }\left( {\frac{1}{{\frac{{{\text{H}}_{{{\text{anode}}}} *{\text{P}}_{{{\text{H}}_{2} {\text{O}}}}^{{{\text{sat}}}} }}{{{\text{P}}_{{{\text{Anode}}}} }}\exp \left( {\frac{{1.622 \times \left( {\frac{{{\text{I}}_{{{\text{cell}}}} }}{{{\text{Area}}}}} \right)}}{{{\text{T}}_{{{\text{Fsft}}}} }}} \right)}}} \right)$$

9 $${\text{the}}\;{\text{ pressure }}\;{\text{of }}\;{\text{oxygen}}\;{\text{ P}}_{{{\text{O}}_{2} }} = 0.5 {\text{H}}_{{{\text{cathode}}}} {\text{P}}_{{{\text{H}}_{2} {\text{O}}}}^{{{\text{sat}}}} { }\left( {\frac{1}{{\frac{{{\text{H}}_{{{\text{cathode}}}} *{\text{P}}_{{{\text{H}}_{2} {\text{O}}}}^{{{\text{sat}}}} }}{{{\text{P}}_{{{\text{cathode}}}} }}\exp \left( {\frac{{4.28 \times \left( {\frac{{{\text{I}}_{{{\text{cell}}}} }}{{{\text{Area}}}}} \right)}}{{1.312{\text{*T}}_{{{\text{Fsft}}}} }}} \right)}}} \right)$$

10 $${\text{V}}_{{{\text{atpil}}}} = {\uptau }_{1} + \tau_{2} {\text{T}}_{{{\text{Fopr}}}} + \left( {{\uptau }_{3} + {\uptau }_{4} } \right){\text{*T}}_{{{\text{Fsft}}}} \times \log ({\text{C}}_{{{\text{O}}_{2} }} + {\text{I}}_{{{\text{cell}}}} )$$

11 $${\text{V}}_{{{\text{Cnte}}}} = - \frac{{{\text{RT}}_{{{\text{Fsft}}}} }}{{\text{N*F}}}{\text{*log}}\left( {1 - \frac{{\text{L}}}{{L_{{{\text{max}}}} }}} \right)$$

12 $${\text{V}}_{{{\text{Ohrls}}}} = {\text{I}}_{{{\text{cell}}}} \times \left( {{\text{R}}_{{{\text{af}}}} + {\text{R}}_{{{\text{cf}}}} } \right)$$

13 $${\text{C}}_{{{\text{O}}_{2} }} = \frac{{{\text{P}}_{{{\text{O}}_{2} }} }}{{51.02{\text{e}}^{4} {*}\exp ( - 498{\text{K}}/{\text{T}}_{{{\text{Fsft}}}} )}}$$

14 $${\text{L}} = \frac{{{\text{one fuel cell }}({\text{I}}_{{{\text{cell}}}} )}}{{\text{one cell Area}}}$$

15 $${\text{R}}_{{{\text{ef}}}} = \frac{{{\upgamma }_{{{\text{ef}}}} {\text{*Q}}}}{{\text{A}}}$$

**Figure 3:**
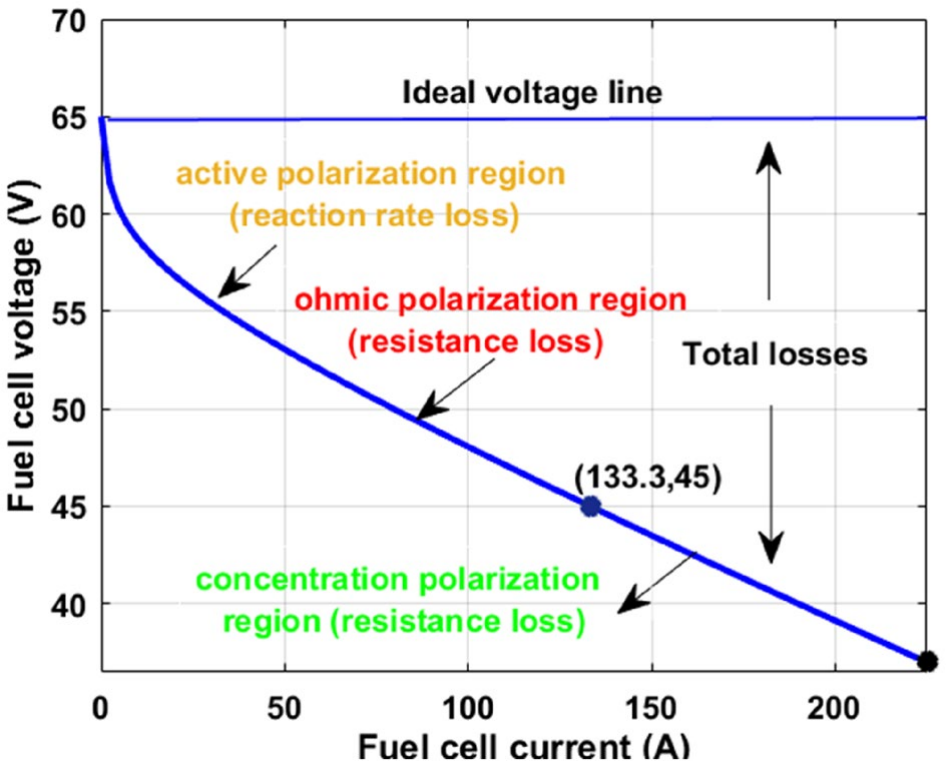
Available peak power point position of the proposed fuel stack network.

Finally, from Eq. (8), and Eq. (9), the utilized single-cell current, and its related area are defined as I_cell_ and A. The available water pressure content in the fuel stack for generating the electricity is ${\text{P}}_{{{\text{H}}_{2} {\text{O}}}}^{{{\text{sat}}}}$. Based on the fuel network equivalent, few major power losses occur in the system which are active, concentrative, plus ohmic losses. The constants ${\uptau }_{1}$, ${\uptau }_{2}$, ${\uptau }_{3}$, and ${\uptau }_{4}$ are the utilized empirical coefficient values. The fuel stack natural gas constant and Faraday constants are defined as “R” and “F”. The anode, and cathode electrodes' effective resistances are R_af_, plus R_cf_. The terms ${\text{R}}_{{{\text{ef}}}}$, Q, and ${\upgamma }_{{{\text{ef}}}}$ are the overall cell resistance, charge, and resistivity of the particular fuel cell. The fuel network produced currents and powers at multiple temperature values conditions are given in Fig. 3. The developed constraints of the fuel stack network are illustrated in Table 1.

**Table 1:** Applied parameters for the development of polymer membrane fuel stack.

Variables of the fuel stack	Values
Peak power appears across the polymer fuel stack (P_MPP_)	6 kW
Peak voltage appears across the polymer fuel stack (V_MPP_)	45.00 V
Peak current appears across the polymer fuel stack (I_MPP_)	133.331 A
Voltage appeared in the fuel network at open-circuited (V_OC_)	64.989 V
Inside the fuel network, the available oxygen pressure parameter	1.189 bar
Inside the fuel network, the available hydrogen pressure parameter	1.822 bar
Overall fuel cells used for the design of fuel network (N)	65
Inside the fuel network, the general rate of airflow (I_pm_)	508.889
Natural gas constant available in the fuel network (R)	85.22189 [J mol^−1^ K^−1^]
Faraday value of the fuel network for various water membranes (F)	93,500.1843 [C mol^−1^]
Overall network chemical oxidization rate value	22.027%
Overall network chemical fuel oxidization rate value	98.667%
Overall hydrogen quantity applied to the system	98.92%
In the fuel network, the effective O_2_ utilization percentage value	61.782%

## Design and exploration of power point tracking controllers

From the literature investigation, all the renewable energy networks needed power point-identifying controllers because of their continuous distortions in the supply of power. Also, the power point identifiers help the renewable systems to work at quick variations of the water membrane values and functioning temperature conditions^21^. Here, the modified continuous step changes adaptive fuzzy logic with grey wolf optimization introduced for functioning the system at peak power point place. The proposed methodology is investigated along with the other conventional power point identifiers which are Adaptive Modified Step Value based P&O (AMSV-P&O), Continuous Step Vary Adaptive based IC (CSVA-IC), Multilayer Variable Step Perceptron Neural Network (MVSPNN), and Modified Step Vary based Radial Basis Functional Network Algorithm (MSV-RBFNA). Here, all five methodologies comprehensive investigation has been carried out in terms of the fuel network production voltage, converted power efficiency, tracking speed of the MPPT, and distortions of the converter power.

### Adaptive modified step value-based P&O controller

Over the past few years, the utilization of the P&O method has been reduced because its drawbacks are less accurate, less efficient, and not applicable for rapid changes in the fuel stack water membrane values. In^22^, the authors studied the adaptive modified step value-dependent P&O methodology applied to the hydrogen fuel stack, and battery charging system for balancing the charging voltage of the battery. This balanced uniform voltage is fed to the bidirectional converter network for running the hydrogen–oxygen vehicle. Here, this controller block collects all the hydrogen stack variables and converter variables for generating the suitable duty pulses to the quadratic-type power converters. In this power point identifier, the present existing power value is associated with the previously stored power values for running the operating point of the fuel stack from the origin place to the MPP position. Suppose, the evaluated value has a negative constant then the functioning point of the fuel stack runs from the right side to the actual power point region. The complete functioning of the adaptive modified step value-dependent P&O methodology is illustrated in Eq. (16) and Eq. (17). From Eq. (16), the parameters ${\text{D}}\left( {{\upgamma } - 1} \right)$, ${\text{D}}\left( {\upgamma } \right)$, ${\text{V}}\left( {{\upgamma } - 1} \right)$, ${\text{V}}\left( {\upgamma } \right)$, ${\text{P}}\left( {{\upgamma } - 1} \right)$, and ${\text{P}}\left( {\upgamma } \right)$ are the power conversion circuit past duty signal, present available duty, past fuel network voltage, actual generated fuel network voltage, and fuel network delivered currents. Here, the term ψ defines the variable step value of the P&O power point identifier.

16 $${\text{D}}\left( {\upgamma } \right) = {\text{D}}\left( {{\upgamma } - 1} \right) + {\psi *}\left| {\frac{{{\text{P}}\left( {\upgamma } \right) - {\text{P}}\left( {{\upgamma } - 1} \right)}}{{{\text{V}}\left( {\upgamma } \right) - {\text{V}}\left( {{\upgamma } - 1} \right)}}} \right|$$

17 $${\text{Duty}}\left( {\upgamma } \right) = {\text{Duty}}\left( {{\upgamma } - 1} \right) - {\psi *}\left( {\left| {\frac{{{\text{P}}\left( {\upgamma } \right) - {\text{P}}\left( {{\upgamma } - 1} \right)}}{{{\text{V}}\left( {\upgamma } \right) - {\text{V}}\left( {{\upgamma } - 1} \right)}}} \right|} \right)$$

### Continuous step vary adaptive based IC technique

The functioning of this continuous step varies adaptively based IC power point identifier is quite equal to the P&O concept. In this, the fuel stack equivalent conductance is utilized for equating the consumer load impedance with the fuel stack network impedance. As a result, the maximum possible fuel network power flows through the converter circuit. The advantages of this methodology are more efficient MPP tracking speed, low dependence on the fuel stack network modeling, heavy flexible operation, better performance when associated with the fractional current methodologies, plus applicability for the electric network systems^23^. However, the development cost of this controller is more when equated with the incremental resistance, closed loop voltage feedback controllers. The updating of fuel stack network conductance is defined in Eq. (18). From Eq. (18), the parameters ${\text{Duty }}\left( {\Phi } \right)$, ${\text{D}}\left( {{\Phi } - 1} \right)$, ${\delta I}$ and ${\delta V}$ are identified as instant converter functioning duty signal, last stored converter duty value, variation in fuel stack current, plus a complete change in the fuel stack network voltage. In this method, the step constant (${\upbeta }$) is varied to reduce the tracking time of the MPP.

18 $${\text{Duty }}\left( {\Phi } \right) = {\text{D}}\left( {{\Phi } - 1} \right) + {\text{D}}_{{{\text{step }}\;{\text{value}}}} {\text{*sign }}\;{\text{indication}}\left( {\frac{{{\delta I}}}{{{\delta V}}} + \frac{{\text{I}}}{{\text{V}}}} \right)$$

19 $${\text{Duty }}\left( {\Phi } \right) = {\text{D}}\left( {{\Phi } - 1} \right) + {\text{D}}_{{{\text{step }}\;{\text{value}}}} {\text{*sign}}\;{\text{indication}}\left( {\frac{{{\delta I}}}{{{\delta V}}} + \frac{{\text{I}}}{{\text{V}}}} \right)$$

20 $${\text{D}}_{{{\text{step}}\;{\text{value}}}} =\upbeta {*}\frac{{{\Delta P}}}{{{\Delta V}}};{\upbeta } = \frac{{{\delta P}}}{{{\delta V}}}$$

### Multilayer variable step perceptron neural network

**Figure 4:**
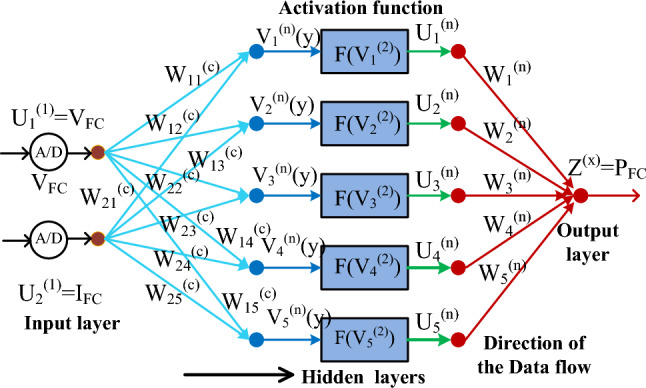
Utilized multiple layers-based perceptron network for fuel stack network.

The conservative controllers are not efficient for the fast changes in the water membrane conditions of the fuel system network. So, the neural methodologies are studied in^24^ for supplying the uniform fuel stack voltage to the automotive system under multiple functioning temperature values. The neural tool is very powerful for solving the multidimensional large space complex nonlinear issues. Neural controller development has been done from the human brain performance. In this network, there are more than three layers are selected for identifying the final nonlinear object. The features of this controller are less statistical training involvement, and the ability to identify the interactions of the all-predictable variables. Also, these neural networks are capable of handling highly complex data, and the ability to sort out all linear and nonlinear modeling capability issues. However, this type of network needs too large an amount of labeled data, more convergence time taking, high complexity in development, and required accurate training data sets. The functioning of the multiplayer neural model is illustrated in Fig. 4. From Fig. 4, the source neuron layer nodes are two which are fuel stack network production voltage, plus converter collected input current. From Fig. 4, the terminologies U, V, plus Z are the neural layers indication. The W_cd_, net_c_, and ΔW are the c and d layers' weight, net value of the source layer, and weight updating between hidden neurons. Finally, y, n, c, d, and G are indicated as source node number, middle node number, load node number, and slope value of the P-I curve.

21 $${\text{V}}_{{\text{c}}}^{\left( 2 \right)} \left( {\text{y}} \right) = \mathop \sum \limits_{{{\text{d}} = 1}}^{2} {\text{W}}_{{{\text{cd}}}}^{\left( 2 \right)} {*}net_{{\text{d}}}^{1} ;\quad {\text{d}} = 1,2,3,4,5 \ldots ,7$$

22 $$net_{{\text{c}}}^{\left( 2 \right)} \left( {\text{y}} \right) = {\text{f}}\left( {{\text{V}}_{{\text{c}}}^{\left( 2 \right)} \left( {\text{y}} \right)} \right)$$

23 $$J^{3} \left( {\text{y}} \right) = \mathop \sum \limits_{{{\text{d}} = 1}}^{5} {\text{W}}_{{\text{d}}}^{\left( 3 \right)} {\text{*L}}_{{\text{d}}}^{\left( 2 \right)}$$

24 $${\text{W}}_{{{\text{cd}}}}^{\left( 2 \right)} = {\text{W}}_{{{\text{cd}}}}^{\left( 2 \right)} + {\Delta W}_{{{\text{cd}}}}$$

25 $${\text{W}}_{{\text{c}}}^{\left( 3 \right)} = {\text{W}}_{{{\text{c}}3}}^{\left( 3 \right)} + {\Delta W}_{{\text{d}}}$$

26 $${\Delta W}_{{{\text{cd}}}} = {\text{G*}}\frac{{\partial {\text{e}}}}{{\partial {\text{W}}_{{{\text{CD}}}}^{\left( 2 \right)} }},\& \quad {\Delta W}_{{\text{d}}} = {\text{G*}}\frac{{\partial {\text{e}}}}{{\partial {\text{W}}_{{\text{d}}}^{\left( 3 \right)} }}$$

27 $${\text{e}} = \frac{1}{2}({\text{P}}_{{{\text{PFC}}}} - {\text{P}}_{{{\text{PFCactual}}}}^{\left( 3 \right)} )^{2}$$

### Modified step vary-based radial basis functional network algorithm

**Figure 5:**
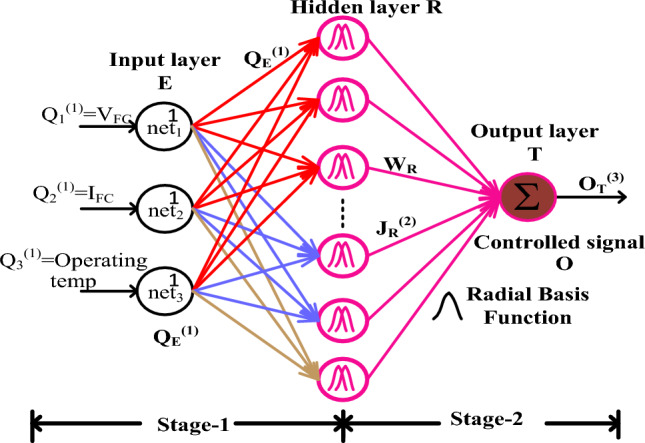
Modified step vary-based radial basis functional network algorithm.

In the multilayer neural model, there are lack of data samples are collected for evaluating the quasi-source interleaved two-phase power converter duty value. Due to the greater number of samples in the network, it needs a proper knowledge candidate for developing and functioning the neural model. Also, it works for a fixed number of source layers and a fixed number of input signals. Moreover, the neural model has a high limiting factor for different types of pattern reorganization^25^. However, the major challenges involved in this neural controller are vanishing gradients, limited labeled data, plus overfitting. These above demerits of conventional models are limited by selecting the radial basis activation function. The schematic workflow of the radial activation function related to MPPT methodology is discussed in Fig. 5. From Fig. 5, the parameters Q, E, and R are the neural layers indication. Also, T, J, and O are illustrated as hidden signals. Finally, j, a, and U are defined as the hidden neurons' interconnected signals. The radial function supplies the fuel stack network error voltage signal to the pulse generator for running the overall network at multiple types of operating water membrane conditions of the fuel stack.

28 $${\text{net}}_{{\text{E}}}^{\left( 1 \right)} = {\text{Q}}_{{\text{E}}}^{1} \left( {\text{j}} \right);\quad {\text{j}} = 1,2,3,4,5,6 \ldots , \ldots {\text{a}}$$

29 $${\text{J}}_{{\text{E}}}^{\left( 1 \right)} \left( {\text{j}} \right) = {\text{f}}_{{\text{E}}}^{\left( 1 \right)} \left( {{\text{net}}_{{\text{E}}}^{\left( 1 \right)} \left( {\text{j}} \right)} \right) = {\text{net}}_{{\text{E}}}^{1} \left( {\text{j}} \right);\quad {\text{j}} = 1,2,3,4,5 \ldots ,{\text{a}}$$

30 $${\text{net}}_{{\text{R}}}^{\left( 2 \right)} \left( {\text{j}} \right) = - ({\upmu } - {\upvarepsilon }_{{\text{R}}} )^{{\text{T}}} {*}\mathop \sum \limits_{{\text{R}}} \left( {{\upmu } - {\upvarepsilon }_{{\text{R}}} } \right)$$

31 $${\text{J}}_{{\text{R}}}^{\left( 2 \right)} \left( {\text{j}} \right) = {\text{f}}_{{\text{R}}}^{\left( 2 \right)} \left( {{\text{net}}_{{\text{R}}}^{\left( 2 \right)} \left( {\text{j}} \right)} \right);\quad {\text{j}} = 1,2,3,4,5, \ldots ,{\text{a}}$$

32 $${\text{net}}_{{\text{U}}}^{\left( 3 \right)} = \mathop \sum \limits_{{\text{R}}} {\text{W}}_{{\text{R}}} {\text{M}}_{{\text{R}}}^{2} \left( {\text{j}} \right);\quad {\text{j}} = 1,2,3,4,5,6 \ldots ,{\text{a}}$$

33 $${\text{N}}_{{\text{T}}}^{\left( 3 \right)} \left( {\text{j}} \right) = {\text{f}}_{{\text{T}}}^{\left( 3 \right)} (\left( {{\text{net}}_{{\text{T}}}^{3} \left( {\text{j}} \right)} \right) = {\text{net}}_{{\text{T}}}^{3} \left( {\text{j}} \right)$$

34 $${\text{error}}\;{\text{value}} = \mathop \sum \limits_{{{\text{a}} = 1}}^{{\text{j}}} \frac{1}{2}\left( {{\text{V}}_{{{\text{ref}}\;{\text{ value}}}} - {\text{V}}_{{{\text{MPP }}\;{\text{value}}}} } \right)$$

### Proposed MCSC adaptive fuzzy logic with GWO MPPT technique

The P&O, multilayer neural models, incremental conductance, and radial networks produce the highly distorted fuel stack energy which may not be fed to the hydrogen vehicle. In this article, the grey wolf concept is introduced along with the adaptive-based fuzzy logic concept. In^26^, the authors investigated the fuzzy concepts for hydrogen vehicle speed control applications. However, the fuzzy has the issues of high difficulty in forming, more complex formulas are required for logical operation and grounding. Also, the fuzzy involves the various categories of membership shapes. Here, the selection of the accurate membership value for the fuel stack system is a very challenging task. Here, the GWO concept is introduced for finding the suitable membership value for the proposed power point identifier. Also, in this hybrid power controlling network, the starting state, a fuzzy methodology is selected for operating the fuel stack network from the origin place to the global MPP place. After that, the optimization methodology is selected for eliminating the oscillations of the load current. Here, the grey wolf does not take a high number of iteration values to reach the optimal nonlinear solution.

**Figure 6:**
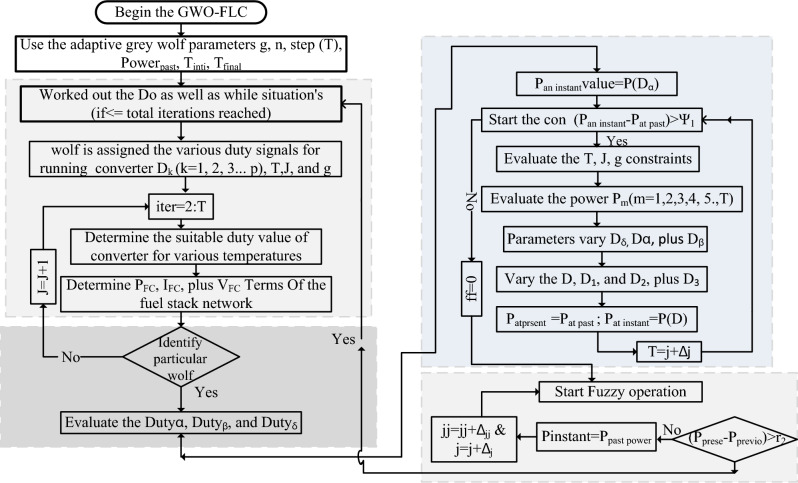
Fuzzy controller fed grey wolf concept for power point identification of fuel network.

The parameters α, β, δ, plus ω are represented as the various wolf leaders. Here, the linear function is applied for varying the distance of the wolf. Based on this liner function, the wolf utilization, and tunning are increased. Also, the searching space and wolf agents should be balanced. All the wolf agents' searching speeds and their associated distances are measured by applying Eq. (37), and Eq. (38). Here, the variable $\overline{{{\text{R}}_{{{\text{Posit}}}} }} \left( {{\text{g}} + 1} \right)$ is the particular wolf place, and its constraints are ${\upvarepsilon }_{1} \in \left( {0,1} \right),{ }\& \upvarepsilon_{2} \in \left( {0,1} \right)$. Finally, the variables T, and j are the particular wolf and its iteration value. The detailed power point identification of fuzzy-based grey wolf technology is discussed in Fig. 6.

35 $$\overline{{{\text{R}}_{{{\text{Posit}}}} }} \left( {{\text{T}} + 1} \right) = {\upvarepsilon }_{1} {*}\overline{{{\text{R}}_{{{\text{Posit}}}} }} \left( {{\text{T}} + 1} \right) + {\upvarepsilon }_{2} {*}\left( {{\text{T}}^{1} - {\text{T}}} \right)$$

36 $${\text{T}}\left( {\text{j}} \right) = {\text{T}}_{{{\text{intial}}}} - \left( {{\text{t}}_{{{\text{int}}}} - {\text{t}}_{{{\text{fin}}}} } \right)\left( {\frac{{{\text{Ma}} - {\text{j}}}}{{{\text{Ma}}}}} \right)$$

37 $$\left| {{\text{Power}}_{{\text{at instant}}} - {\text{Power}}_{{\text{at past}}} } \right| \le \Upsilon_{1}$$

38 $$\left| {{\text{Power}}_{{\text{at instant}}} - {\text{Power}}_{{\text{at past}}} } \right| \ge \Upsilon_{1}$$

## Development of new wide power DC–DC converter

The major difficulty of handling fuel stack networks is excessive supply current which is very dangerous for hydrogen-dependent vehicles. So, the DC–DC converter circuits are placed near the fuel stack network to eliminate the over-current flow of the fuel stack network. In^27,28^, the researchers studied the isolated technology for the power conversion of the hydrogen-dependent vehicle. However, the isolated power transformation technologies needed more additional components like power rectifiers, plus step-up transformers. Due to this condition, the entire fuel stack network implementation size and cost are improved. Here, a new universal power supply DC–DC circuit is used to enhance the power transformation capability of the fuel source system. The proposed circuit involves the 5-capacitors (C_y_, C_u_, C_i_, C_p_, and C_0_), 3-didoes (D_y_, D_u_, and D_i_), and 3-inductors (L_y_, L_u,_ and L_i_). Here, a Metal Oxide Semiconductor Field Effect Transistor (MOSFET) is selected as a power switch for high-voltage transmission of the network. The filter circuit is placed near the fuel network by using the components C_y_, D_u_, D_i_, L_u_, L_i_, C_o_, and C_p_ respectively.

### Working state of converter circuit-I

**Figure 7:**
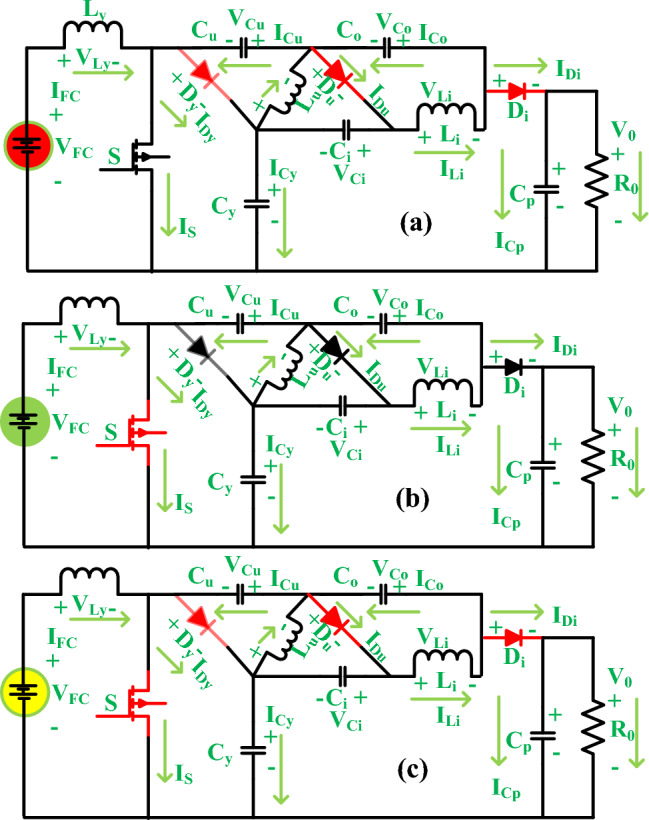
Wide power transformation converter, (**a**) MOSFET ON and diodes are OFF, (**b**) diodes are ON and MOSFET is OFF, and (**c**) all power semiconductor elements are in an ideal state.

The working stages of the converter are discussed in Table 2. From Table 2, the devices D_y_, D_u_, and D_i_ are moving from the working stage to the ideal state. The selected new converter circuit is mentioned in Fig. 7a, and the MOSFET starts working to provide a uniform voltage to the load. From Fig. 7a, the highlighted filter elements captured the fluctuated converter currents and fuel network voltages for enhancing the efficiency of the overall network. Here, the charged and discharged capacitive components currents and voltages are identified as I_Cy_crgn_, I_Cu_crgn_, I_Ci_crgn_, I_Co_crgn_, and I_Cp_crgn_, V_Cy_crgn_, V_Cu_crgn_, V_i_crgn_, V_Co_crgn_, and V_Cp_crgn_, I_Cy_drgng_, I_Cu_ drgng_, I_Ci_ drgng_, I_Co_ drgng_, and I_Cp_ drgng_, V_Cy_ drgng_, V_Cu_ drgng_, V_i_ drgng_, V_Co_ drgng_, and V_Cp_ drgng_ respectively. Here, the inductors capture the fuel stack network energy for supplying the energy to the standalone system. Here, the elements C_u_, and C_i_ collect the source energy, plus the elements C_y_, C_i_, and C_p_ deliver the power to the consumer as discussed in Eq. (32), and Eq. (39).

39 $$\left\{ {\begin{array}{*{20}l} {{\text{I}}_{{{\text{Cu}} - {\text{crgn}}}} = {\text{I}}_{{{\text{Cy}} - {\text{drgng}}}} = {\text{I}}_{{{\text{Lu}}}} + {\text{I}}_{{{\text{Li}}}} } \hfill \\ {{\text{I}}_{{{\text{Ci}} - {\text{drgng}}}} = {\text{I}}_{{{\text{Co}} - {\text{crgn}}}} = {\text{I}}_{{{\text{Li}}}} } \hfill \\ {{\text{I}}_{{{\text{C}}0 - {\text{disg}}}} = {\text{I}}_{0} } \hfill \\ \end{array} } \right.$$

40 $$\left\{ {\begin{array}{*{20}l} {{\text{V}}_{{{\text{Ly}}}} = {\text{V}}_{{{\text{FC}}}} } \hfill \\ {{\text{V}}_{{{\text{Lu}}}} = {\text{V}}_{{{\text{Cy}}}} - {\text{V}}_{{{\text{Cu}}}} } \hfill \\ {{\text{V}}_{{{\text{Li}}}} = {\text{V}}_{{{\text{Cy}}}} + {\text{V}}_{{{\text{Ci}}}} - {\text{V}}_{{{\text{Co}}}} - {\text{V}}_{{{\text{Cu}}}} } \hfill \\ \end{array} } \right.$$

**Table 2:** The working state of the power converter circuit.

	I state CCS and DCS	II state CCS and DCS	3rd state DCS
S	Working	Not working	Not working
D_Y_	Not working	Working	Not working
D_U_	Not working	Working	Not working
D_I_	Not working	Working	Not working

### Working state of converter circuit-II

In this stage, the converter utilized three diodes to move in the running stage for delivering the voltage to the load. For analyzing the Continuous Conduction State (CCS), and Discontinuous Conduction State (DCS) of the proposed power converter circuit, a few approximations are utilized which are selected values for the passive components are more, and second all semiconductor elements are ideal. Finally, the passive components' internal impedances are neglected. The values used for the development of passive elements are very high to run the converter circuit in CCS and DCS operations. In this state, the elements C_u_, and C_i_ give the power to the consumer, and the elements C_y_, C_i_, and C_p_ capture the overall available power as mentioned in Eq. (41), and Eq. (42). The running of the converter circuit in this state illustrate in Fig. 7b. From Fig. 7b, it has been indicated that the consumer power is constant irrespective of the changes in the fuel network water membrane conditions.

41 $$\left\{ {\begin{array}{*{20}l} {{\text{I}}_{{{\text{Cu}} - {\text{drgng}}}} = {\text{I}}_{{{\text{Co}} - {\text{drgng}}}} + {\text{I}}_{{{\text{Li}}}} + {\text{I}}_{{{\text{Li}} - {\text{crgn}}}} - {\text{I}}_{{{\text{Lu}}}} } \hfill \\ {{\text{I}}_{{{\text{Cy}} - {\text{crgn}}}} = - {\text{I}}_{{{\text{Cu}} - {\text{drgn}}}} + {\text{I}}_{{{\text{Ly}}}} - {\text{I}}_{{{\text{Lu}}}} + {\text{I}}_{{{\text{Ci}} - {\text{crgn}}}} } \hfill \\ {{\text{I}}_{{{\text{Cp}} - {\text{crgn}}}} = {\text{I}}_{{{\text{Co}} - {\text{drgn}}}} + {\text{I}}_{{{\text{Li}}}} - {\text{I}}_{0} } \hfill \\ \end{array} } \right.$$

42 $$\left\{ {\begin{array}{*{20}l} {{\text{V}}_{{{\text{Ly}}}} = {\text{V}}_{{{\text{FC}}}} } \hfill \\ {{\text{V}}_{{{\text{Lu}}}} = - {\text{V}}_{{{\text{Cu}}}} = - {\text{V}}_{{{\text{Ci}}}} } \hfill \\ {{\text{V}}_{{{\text{Li}}}} = {\text{V}}_{{{\text{Cy}}}} + {\text{V}}_{{{\text{Ci}}}} - {\text{V}}_{{{\text{Co}}}} - {\text{V}}_{{{\text{Cu}}}} } \hfill \\ \end{array} } \right.$$

### Working state of converter circuit-III

**Figure 8:**
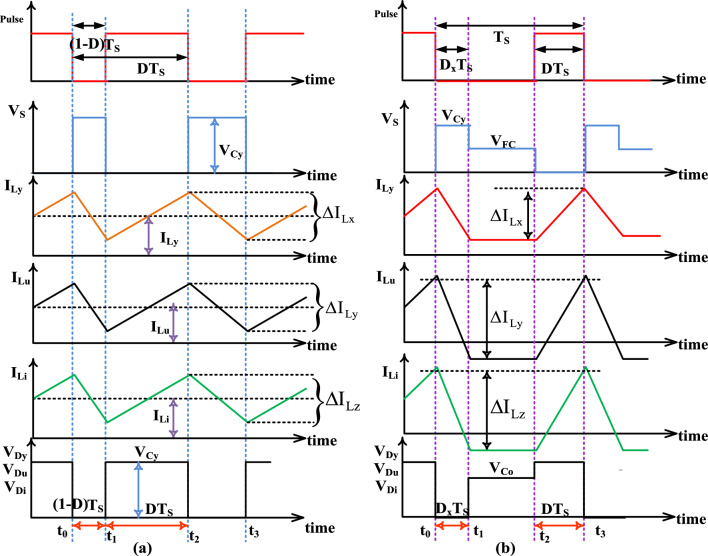
(**a**) Converter circuit generated waveforms under CCS, and (**b**) DCS power converter circuit waveforms.

Here, all the power electronic semiconductor devices are in ideal condition then the energy storage elements only give the power production to the resistor. In this mode, the applied activating MOSFET voltage source is much less, and the inductor's average current is zero. Finally, the peak overvoltage’s of the all-passive inductors are equal to zero. The working states of the devices under this condition are mentioned in Fig. 7c. The converter structure generated switching pulses at CCS, plus DCS are defined in Fig. 8a, b.

43 $${\text{I}}_{{{\text{Lymin}}}} + {\text{I}}_{{{\text{Lumin}}}} + {\text{I}}_{{{\text{Limin}}}} = 0$$

44 $${\text{V}}_{{{\text{Ly}}}} = {\text{V}}_{{{\text{Lu}}}} = {\text{V}}_{{{\text{Li}}}} = 0$$

### Evaluation of converter gain voltage and its comprehensive analysis

The voltage conversion value of the converter is obtained by selecting the state-I, and State-II modes of the operation of the DC–DC converter. From Fig. 8a, and Eq. (35), the steady-state voltage gain of the DC–DC circuit is evaluated by utilizing Eq. (44). Similarly, from Eq. (44), and Fig. 8b, the voltage appeared across power diodes and switches is obtained by using Eq. (47). Here, the voltage appeared across each power diode is equal to V_Cy_. By utilizing Eq. (41), and (42), the voltage appeared across the switch, and the power diode is obtained in terms of voltage gain which is given in Eq. (48).

45 $${\text{V}}_{{{\text{Cu}}}} = {\text{V}}_{{{\text{Co}}}} = {\text{V}}_{{{\text{Ci}}}} = \frac{{\text{D}}}{{\left( {1 - {\text{D}}} \right)}}*{\text{V}}_{{{\text{FC}}}}$$

46 $${\text{V}}_{{{\text{Cy}}}} = \frac{1}{{\left( {1 - {\text{D}}} \right)}}{\text{*V}}_{{{\text{FC}}}} { }\left( {{\text{fuel }}\;{\text{stack }}\;{\text{voltage}}} \right)$$

47 $${\text{V}}_{0} \left( {{\text{converter }}\;{\text{output }}\;{\text{voltage}}} \right) = \frac{{1 + 2{\text{D}}}}{{1 - {\text{D}}}}{\text{V}}_{{{\text{FC}}}}$$

48 $${\text{Gain}}_{{{\text{CCS}}}} = \frac{{{\text{V}}_{0} \left( {{\text{output}}} \right)}}{{{\text{V}}_{{{\text{FC}}}} }} = \frac{{1 + 2{\text{D}}}}{{1 - {\text{D}}}}$$

49 $$\left\{ {\begin{array}{*{20}l} {{\text{V}}_{{\text{S}}} = {\text{V}}_{{\text{D}}} \left( {Diode} \right) = {\text{V}}_{{{\text{FC}}}} *\frac{1}{{1 - {\text{D}}}}{\text{V}}_{{{\text{Cy}}}} } \hfill \\ {{\text{V}}_{{{\text{Dy}}}} = {\text{V}}_{{{\text{Du}}}} = {\text{V}}_{{{\text{Di}}}} = {\text{V}}_{{\text{D}}} \left( {diode} \right)} \hfill \\ \end{array} } \right.$$

50 $${\text{V}}_{{{\text{Diode}}}} = {\text{V}}_{{\text{S}}} = \frac{{2 + {\text{Gain}}\;{\text{ of}}\;{\text{ DC}} - {\text{DC}}_{{{\text{CCS}}}} }}{{3{\text{*Gain }}\;{\text{of }}\;{\text{DC}} - {\text{DC}}_{{{\text{CCS}}}} }}{\text{*V}}_{0}$$

51 $${\text{I}}_{{{\text{Lu}}}} = {\text{I}}_{{{\text{Li}}}} = {\text{I}}_{{0{\text{ut}}}}$$

52 $${\text{I}}_{{{\text{Ly}}}} = \frac{{1 + 2{\text{D}}}}{{1 - {\text{D}}}}*{\text{I}}_{0} = {\text{Gain}}\;{\text{ of}}\;{\text{ DC}} - {\text{DC}}_{{{\text{CCS}}}} {\text{I}}_{0}$$

53 $$\left\{ {\begin{array}{*{20}l} {{\text{I}}_{{{\text{Cu}} - {\text{crng}}}} = 2*{\text{I}}_{{0{\text{ut}}}} } \hfill \\ {{\text{I}}_{{{\text{Cu}} - {\text{drng}}}} = 2{\text{I}}_{{0{\text{ut}}}} *\frac{{\text{D}}}{{1 - {\text{D}}}} = \frac{{{\text{Gain}}\;{\text{ of }}\;{\text{DC}} - {\text{DC}}_{{{\text{CCs}}}} - 1}}{3}2{\text{*I}}_{0} } \hfill \\ \end{array} } \right.$$

54 $$\left\{ {\begin{array}{*{20}l} {{\text{I}}_{{{\text{Co}} - {\text{crng}}}} = {\text{I}}_{{0{\text{ut}}}} } \hfill \\ {{\text{I}}_{{{\text{Co}} - {\text{drng}}}} = {\text{I}}_{{0{\text{ut}}}} \frac{{\text{D}}}{{1 - {\text{D}}}} = \frac{{{\text{Gain}}_{{{\text{CCs}}}} - 1}}{3}{\text{*I}}_{0} } \hfill \\ \end{array} } \right.$$

55 $$\left\{ {\begin{array}{*{20}l} {{\text{I}}_{{{\text{Ci}} - {\text{crng}}}} = 2*{\text{I}}_{0} *\frac{{\text{D}}}{{1 - {\text{D}}}} = 2*{\text{I}}_{0} *\frac{{{\text{Gain}}_{{{\text{CCs}}}} - 1}}{3}} \hfill \\ {{\text{I}}_{{{\text{Ci}}_{{{\text{drng}}}} }} = 2*{\text{I}}_{0} } \hfill \\ \end{array} } \right.$$

56 $$\left\{ {\begin{array}{*{20}l} {{\text{I}}_{{{\text{Cy}} - {\text{crng}}}} = {\text{I}}_{{0{\text{ut}}}} *\frac{{\text{D}}}{{1 - {\text{D}}}} = \frac{{{\text{Gain}}_{{{\text{CCs}}}} - 1}}{3}{\text{*I}}_{{0{\text{ut}}}} } \hfill \\ {{\text{I}}_{{{\text{Cy}} - {\text{drng}}}} = {\text{I}}_{{0{\text{ut}}}} } \hfill \\ \end{array} } \right.$$

57 $$\left\{ {\begin{array}{*{20}l} {{\text{I}}_{{{\text{Cp}} - {\text{crng}}}} = {\text{I}}_{{0{\text{ut}}}} *\frac{{\text{D}}}{{1 - {\text{D}}}} = \frac{{{\text{Gain}}_{{{\text{CCs}}}} - 1}}{3}{\text{*I}}_{{0{\text{ut}}}} } \hfill \\ {{\text{I}}_{{{\text{C}}0 - {\text{disg}}}} = {\text{I}}_{0} } \hfill \\ \end{array} } \right.$$

The currents of power diodes and switches are evaluated by applying the Fig. 7b, plus (c).

58 $${\text{I}}_{{\text{S}}} = {\text{I}}_{{{\text{Ly}}}} + {\text{I}}_{{{\text{Lu}}}} + {\text{I}}_{{{\text{Li}}}} = {\text{I}}_{{0{\text{ut}}}} *\frac{3}{{1 - {\text{D}}}}$$

59 $${\text{I}}_{{{\text{Dy}}}} = {\text{I}}_{{{\text{Ly}}}} - {\text{I}}_{{{\text{Cu}}_{{{\text{drng}}}} }} = {\text{I}}_{{0{\text{ut}}}} *\frac{1}{{1 - {\text{D}}}}$$

60 $${\text{I}}_{{{\text{Du}}}} = {\text{I}}_{{{\text{Lu}}}} + {\text{I}}_{{{\text{Cu}} - {\text{crng}}}} - {\text{I}}_{{{\text{Co}} - {\text{drng}}}} = {\text{I}}_{{0{\text{ut}}}} *\frac{1}{{1 - {\text{Duty}}}}$$

61 $${\text{I}}_{{{\text{Di}}}} = {\text{I}}_{{{\text{Co}} - {\text{drng}}}} = {\text{I}}_{{0{\text{ut}}}} \left( {1 - \frac{3}{{1 - {\text{D}}}}} \right)$$

The currents that appeared across the power diodes in terms of voltage conversion are given in Eq. (62), and Eq. (63).

62 $${\text{I}}_{{\text{S}}} = \left( {{\text{Gain}}_{{{\text{CCS}}}} + 2} \right){\text{*I}}_{{0{\text{ut}}}}$$

63 $${\text{I}}_{{{\text{Dy}}}} = {\text{I}}_{{{\text{Du}}}} = {\text{I}}_{{{\text{Di}}}} = \left( {\frac{{{\text{Gain}}_{{{\text{CCs}}}} + 2}}{3}} \right)*{\text{I}}_{{0{\text{ut}}}}$$

Finally, the room mean square current value that appears across each semiconductor is derived as,

64 $${\text{I}}_{{{\text{S}}_{{{\text{RMS}}}} }} = \sqrt {\left( {{\text{Gain}}_{{{\text{CCs}}}} + 2} \right)\left( {{\text{Gain}}_{{{\text{CCs}}}} - 1} \right)} {\text{I}}_{{0{\text{ut}}}}$$

65 $${\text{I}}_{{{\text{Dy}} -_{{{\text{RMS}}}} }} = {\text{I}}_{{{\text{Du}} -_{{{\text{RMS}}}} }} = {\text{I}}_{{{\text{Di}} -_{{{\text{RMS}}}} }} = \sqrt {\frac{{{\text{Gain}}_{{{\text{CCs}}}} + 2}}{3}} {\text{I}}_{{0{\text{ut}}}}$$

66 $${\text{I}}_{{{\text{Cu}} -_{{{\text{RMS}}}} }} = {\text{I}}_{{{\text{Cy}} -_{{{\text{RMS}}}} }} = \sqrt[2]{{\frac{{{\text{Gain}}_{{{\text{CCs}}}} - 1}}{3}}}{\text{I}}_{{0{\text{ut}}}}$$

67 $${\text{I}}_{{{\text{Ci}} -_{{{\text{RMS}}}} }} = {\text{I}}_{{{\text{Co}} -_{{{\text{RMS}}}} }} = {\text{I}}_{{{\text{Cp}} -_{{{\text{RMS}}}} }} = \sqrt {\frac{{{\text{Gain}}_{{{\text{CCs}}}} - 1}}{3}} {\text{I}}_{{0{\text{ut}}}}$$

## Proposed system results analysis

A 6 kW polymer membrane fuel stack network is utilized in this article for the study of the fuzzy-based grey wolf concept. The maximum possible generated voltage in the fuel network is 45 V, and the current produced in the network is 133.33 A. So, the current of the fuel stack is higher. To increase the voltage level of the fuel network, a wide supply power unique switch converter circuit is used to remove the fluctuations in the load voltage. The fuel source side inductor L_y_ value is 100 mH which helps to smooth the supply power from the quick variations of the fuel stack temperature. Also, it captures the fuel stack energy under the MOSFET functioning state. Similarly, the utilized load inductive (L_u_, & L_i_) elements values are 120 mH, and 100 mH. These elements support the peak load current flow demand and reduce the heating conduction losses of the system. Finally, the values used for the converter design capacitors (C_y_, C_u_, C_i_, C_p_, and C_0_) are 20 µF. The main use of these capacitors is removing the peak voltages that appear across the power semiconductor devices. Here, the entire fuel stack network is analyzed at a static 322 Kelvin temperature. Also, the fuel stack functioning point identification is observed at 322 K, 302 K, and 342 K temperature conditions.

### Analysis of Wide Power DC–DC Circuit at 322 K

**Figure 9:**
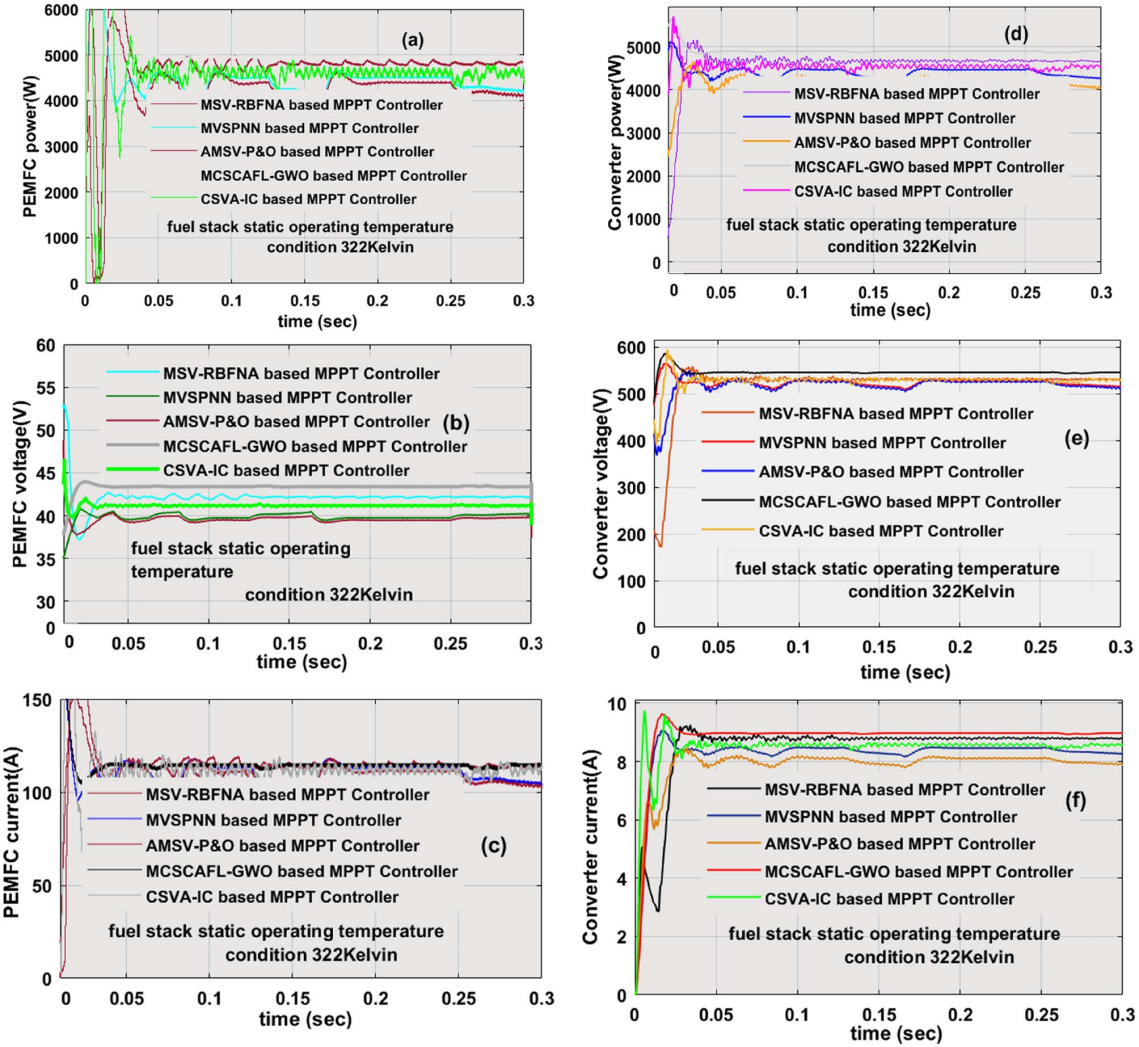
At constant temperature (322 Kelvin), (**a**) fuel network power, (**b**) fuel network voltage, (**c**) fuel current, (**d**) converter network power, (**e**) converter network voltage, and (**f**) converter current.

Here, the fuel stack network performance at 322 K functioning temperature is studied by applying the various power point identifiers. The evaluated fuel network power, fuel stack voltage, fuel supply current, power converter available power, load voltage, and resistor currents by integrating the AMSV-P&O, CSVA-IC, MVSPNN, MSV-RBFNA, and MCSCAFLGWO power point identifiers are 4541.9W, 38.95V, 116.6A, 4378.89W, 520.497V, 8.4129A, 4691.6W, 40.13V, 116.9A, 4575.01W, 515.0182V, 8.8832A, 4687.8W, 39.69V, 118.1A, 4589.07W, 514.9777V, 8.9112A, 4882.6W, 44.63V, 109.4A, 4820.11W, 540.310V, 8.9210A, 4880.7W, 44.04V, 110.8A, 4821.32W, 540.919V, and 8.9132A respectively. Here, the maximum possible power extraction efficiency of the fuel stack network by applying the CSVA-IC, MVSPNN, and MSV-RBFNA power point identifiers are 97.513%, 97.892%, and 98.782%. The investigation of various MPPT methods and their tracking time of the MPP are illustrated in Table 3. The fuel network supply powers, voltages, and fuel stack output currents are discussed in Fig. 9a–c and its associated converter load power, resistive voltage, and currents are defined in Fig. 9d–f. From Fig. 9f, the converter utilized power by applying the neural controller completely depending on the selection of the fuel stack network. However, the AMSV-P&O and CSVA-IC methods work by using the nonlinear curves of the source. Also, these controllers' design is very easy because of the low amount of sensing devices used in the network.

**Table 3:** Evaluated values of the polymer membrane-based power point identifiers at quick change of temperature conditions.

Controller Type	Current of cell	Voltage of cell	Power of cell	DC–DC Current	DC–DC Voltage	DC–DC Power	Working efficiency	MPP time	Cell dependent	Distortions	Complexity
The functioning of the overall fuel stack network at utilizing temperature is 322 K											
AMSV-P&O	116.6A	38.95 V	4541.9W	8.4129A	520.497 V	4378.89W	96.4123%	0.0368 s	Never	More	Very low
CSVA-IC	116.9A	40.13 V	4691.6W	8.8832A	515.0182 V	4575.01W	97.513%	0.03712 s	Never	More	Very low
MVSPNN	118.1A	39.69 V	4687.8W	8.9112A	514.9777 V	4589.07W	97.892%	0.0481 s	Complete	More	Medium
MSV-RBFNA	109.4A	44.63 V	4882.6W	8.9210A	540.310 V	4820.11W	98.782%	0.0556 s	Complete	Medium	Medium
MCSCAFLGWO	110.8A	44.04 V	4880.7W	8.9132A	540.919 V	4821.32W	98.783%	0.0612 s	Partially	Low	Medium
The functioning of the overall fuel stack network at utilizing temperature is 302 K											
AMSV-P&O	104.5A	41.44 V	4331.5W	8.24510A	511.791 V	4219.77W	97.4231%	0.0353 s	Never	More	Very low
CSVA-IC	106.7A	40.54 V	4326.6W	8.32108A	507.2766 V	4221.09W	97.561%	0.03618 s	Never	More	Very low
MVSPNN	104.8A	40.86 V	4282.4W	8.33198A	507.585 V	4229.19W	98.756%	0.0401 s	Complete	More	Medium
MSV-RBFNA	104.2A	41.06 V	4278.7W	8.34691A	506.9265 V	4231.27W	98.891%	0.0471 s	Complete	Medium	Medium
MCSCAFLGWO	103.6A	41.21 V	4269.5W	8.3561A	507.2809 V	4238.89W	99.281%	0.0591 s	Partially	Low	Medium
The functioning of the overall fuel stack network at utilizing temperature is 342 K											
AMSV-P&O	122.9A	40.36 V	4961.1W	9.0012A	539.4214 V	4855.44W	97.896%	0.032 s	Never	More	Very low
CSVA-IC	119.3A	43.99 V	5248.7W	9.18270A	560.824 V	5149.88W	98.117%	0.0318 s	Never	More	Very low
MVSPNN	119.8A	44.65 V	5350.0W	9.2508A	568.4059	5258.21W	98.283%	0.0310 s	Complete	More	Medium
MSV-RBFNA	120.8A	43.84 V	5295.9W	9.28612A	566.8072 V	5263.44W	99.387%	0.029 s	Complete	Medium	Medium
MCSCAFLGWO	121.2A	44.22 V	5359.9W	9.2992A	573.0482 V	5328.89W	99.421%	0.0285 s	Partially	Low	Medium

### Analysis of Wide Power DC–DC Circuit at 322 K, 302 K, and 342 K

**Figure 10:**
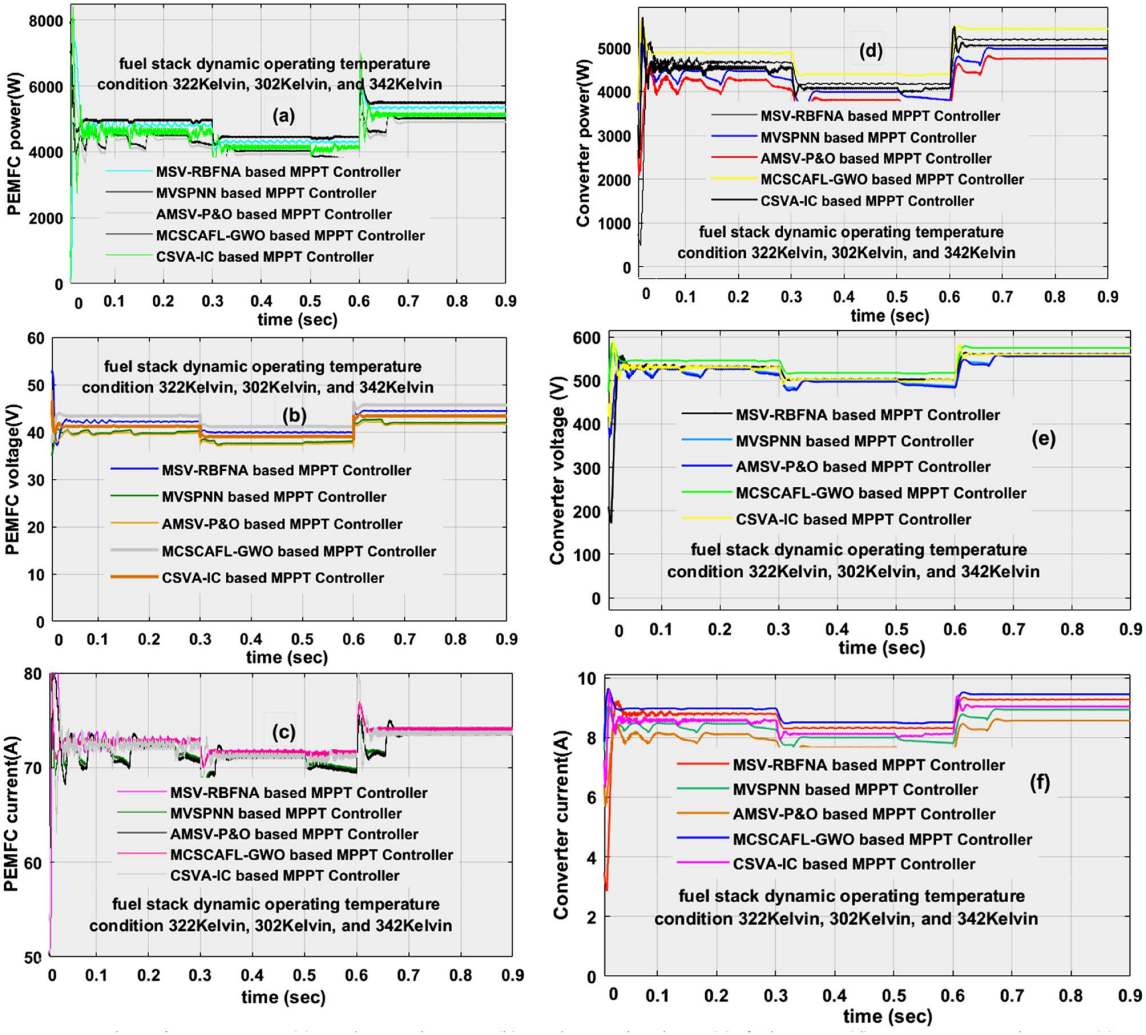
At dynamic temperature, (**a**) fuel network power, (**b**) fuel network voltage, (**c**) fuel current, (**d**) converter network power, (**e**) converter network voltage, and (**f**) converter current.

The maximum peak voltage extraction from the fuel network by selecting the quick changes in the functioning temperature conditions of the fuel stack is a highly difficulty task. So, in this article, a combination of the grey wolf concept, and fuzzy technology is introduced to find the exact position of the fuel network MPP place. The generated source power waveforms, fuel system available voltage waveforms, current waveforms, converter produced power, converter boosted voltage, and current waveforms are illustrated in Fig.10a–f. At 302K, from Fig.10, the evaluated supply currents, voltage, fuel network power, converter optimized current, load voltage, and converter powers by applying the AMSV-P&O, CSVA-IC, MVSPNN, MSV-RBFNA, and MCSCAFLGWO MPPT methods are 104.5A, 41.44V, 4331.5W, 8.24510A, 511.791V, 4219.77W, 106.7A, 40.54V, 4326.6W, 8.32108A, 507.2766V, 4221.09W, 104.8A, 40.86V, 4282.4W, 8.33198A, 507.585V, 4229.19W, 104.2A, 41.06V, 4278.7W, 8.34691A, 506.9265V, 4231.27W, 103.6A, 41.21V, 4269.5W, 8.3561A, 507.2809V, and 4238.89W respectively. Also, these controllers power point identifying time, and its associated efficiencies under rapid variation of fuel stack temperature (302K) conditions are 0.0353sec. 97.4231%, 0.03618sec, 97.561%, 0.0401sec, 98.756%, 0.0471sec, 98.891%, 0.0591sec, and 99.281% respectively. At 342K, the system-investigated values are discussed in detail in Table 3.

## Development of experimental converter prototype

**Figure 11:**
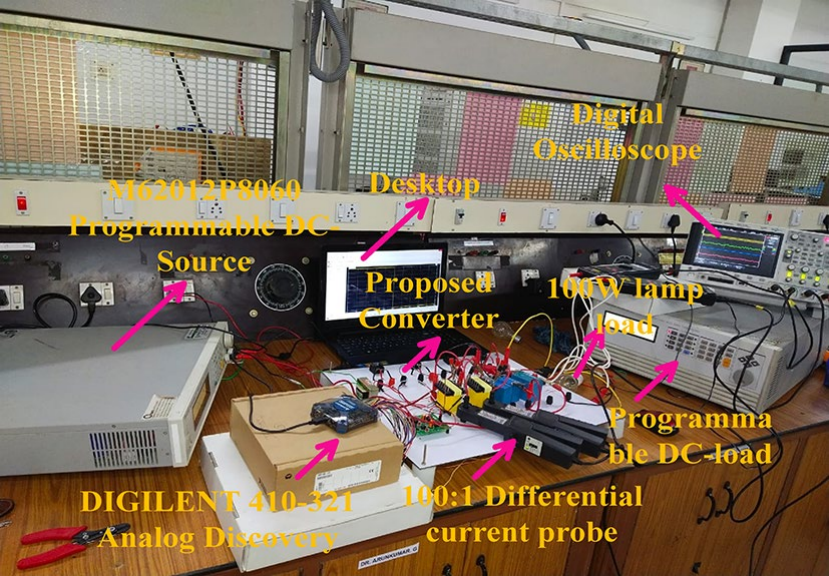
Introduced power conversion circuit testing network.

**Figure 12:**
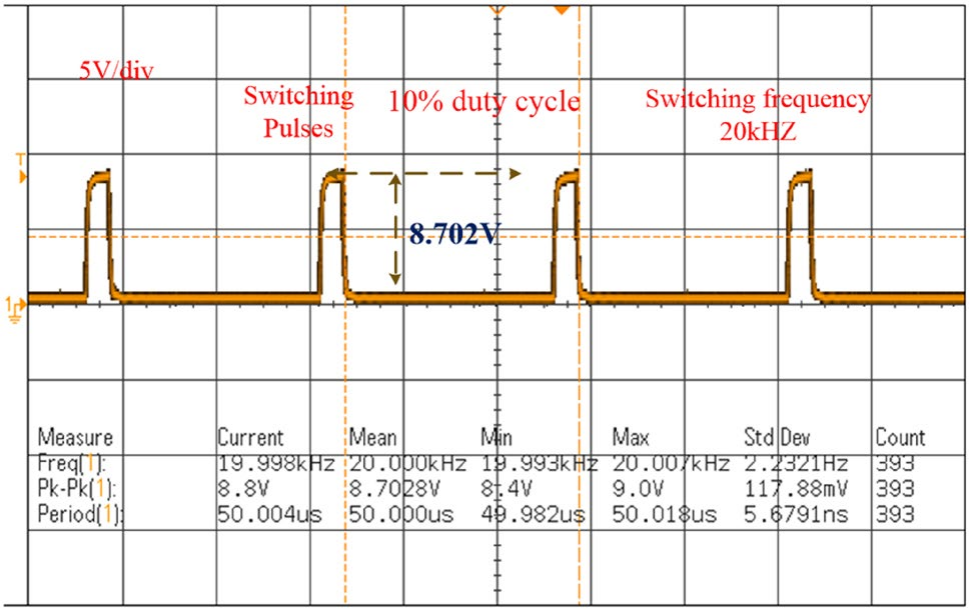
Utilized power conversion circuit duty signal waveform.

**Figure 13:**
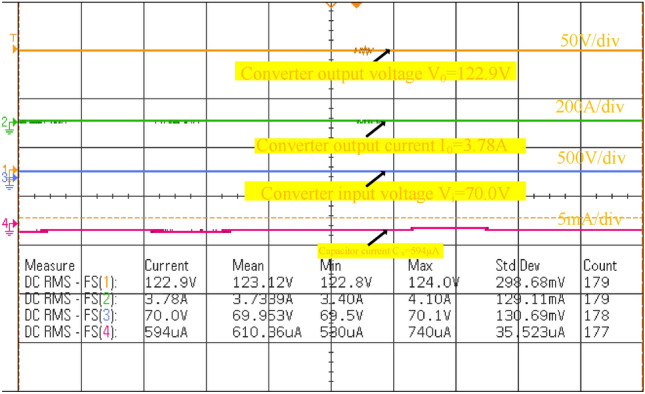
Identified power conversion network available waveforms.

Here, the introduced power converter testing has been made by selecting the programmed M55024P8058 direct voltage source. Also, the analog discovery component is utilized for the pulse generation of the power conversion network. From Fig.11, a 230V power supply is converted to 12V by selecting the step-down transformer to run the TLP-450 driver technology. This device helps the power conversion circuit from fast changes in the grid voltage. Also, it behaves as an isolator in the middle of the transformer plus a power switch. Here, TLP consumes the 0.07A current, and 5V voltage to activate the MOSFET. The Gallium Arsenide helps the photodetector for monitoring the switching stages of the network. The power conversion system investigation is carried out by applying the 0.1 duty value of the network as illustrated in Fig.12, and its associated functioning frequency is up to 20kHZ. The supplied source voltage to this switch is 8.702V and its voltage production rate is uniform. The tested power conversion circuit values are similar to the simulation study. Finally, from Fig.13, the converter circuit enhanced the voltage level from 70.0V to 122.9V at 0.1 duty signal.

## Conclusion

The fuzzy-based grey wolf methodology is developed by selecting the MATLAB tool for the fuel stack network to obtain the maximum possible load voltage of the system. In the 1st objective, the PEMFS device is used in the proposed network because its features are more power density, quick functioning, more reliability, the capability to function at very low-temperature levels, less expensive, and longer lifetime. In the 2nd stage, the new power conversion circuit is developed to reduce the fuel stack supply current thereby optimizing the entire network power losses. The proposed DC–DC conversion circuit features are more voltage conversion value, less passive components usage, wide voltage source, better dynamic performance, and high functioning efficiency. Finally, the hybrid MPPT method helps identify the functioning point of the fuel network. This method provides good MPP tracing speed and works more efficiently when associated with the other power point identifiers.

## Data Availability

The datasets used and/or analyzed during the current study are available from the corresponding author upon reasonable request.
